# COVID-19, *Contagion,* and Vaccine Optimism

**DOI:** 10.1007/s10912-021-09677-3

**Published:** 2021-02-15

**Authors:** Kelly McGuire

**Affiliations:** Departments of English and Gender & Women’s Studies, Trent University, Peterborough, Ontario Canada

**Keywords:** Medical humanities, COVID-19, Vaccine, Immunity, Film, Popular culture, Vaccine hesitancy

## Abstract

Steven Soderbergh’s *Contagion* (2011) positions the vaccine as the end point of the arc of ​pandemic, marking both the containment of an elusive virus and ​the resumption of a life not fundamentally different from ​before the disease outbreak. ​The film reinforces the ​assumption that a pandemic will awaken ​all of us to the urgency of vaccination​, persuading us to put aside our reservations and anxieties ​and the idea that compliance is the inevitable outcome of quarantine. This article explores how pro-vaccination cultural products ​such as *Contagion* might in fact undermine public health efforts by promoting a false narrative, which simplifies the kind of vaccination campaign necessary for herd immunity to develop. An ethic of sacrifice and selflessness drives the public health messaging of the film but leaves intact certain individualistic tropes and plague narrative scapegoating tendencies, while the framing of the vaccine as “gift” takes it out of the realm of medical science altogether.

Although some might believe that the conditions of pandemic create compliance, from the outset of coronavirus lockdowns in the West there were strong indications that this assumption might not in fact be tenable.^1^ In the spring of 2020, Emily Harrison and Julia Wu and cautioned against the “rush of vaccine optimism…pervading present discourse around the COVID-10 epidemic” and called for the re-imagination of the culture of public health and the meaning of vaccine safety regulations” (325). Agreeing with Harrison and Wu’s caveat, this essay argues that an interrogation of culture itself—specifically popular culture—is vital for addressing the reality of vaccine hesitancy that this overly optimistic discourse often ignores. The anti-vaccination movement has gained momentum in recent years, owing in part to the fact that it took *The Lancet* ten years to retract Andrew Wakefield’s fraudulent study linking the MMR vaccine to autism.^2^ In this time, anti-vaccination discourse proliferated on social media and across platforms, accommodating diverse worldviews whereas, as Heidi Larson of the Vaccine Confidence Project observes, pro-vaccination conversations tended to be insular and inward looking, reverberating through the echo chambers of platforms like Facebook rather than reaching out to those with divergent opinions.^3^ Building on this well-established foundation of skepticism, opposition to a SARS-CoV-2 vaccine hardened long before one emerged as a viable candidate, a position that will only become more intractable over time unless a meaningful public conversation can take place. Many medical authorities insisted in the spring of 2020 on the urgency of addressing vaccine hesitancy while vaccines were under development even if the date one would become widely available remained elusive.^4^

At that time, the politics of prophylactic mask-wearing served as a grim harbinger of the individual freedom debates sure to mobilize anti-vaccination campaigns. Dismissed as a political symbol of “the anarchic and radical left” by certain politicians, the mask is now symbolically linked to the vaccine with resistance to face covering prefiguring the struggles to come. Both the mask and the vaccine act as a barrier, one not only offering a degree of protection but also seeking to reduce the possibility of infecting others. But a vaccine is much more difficult to visualize than a mask, figured metonymically through the physician’s syringe and off-putting language like the British “jab” and acting mysteriously in invisible ways on the body, its ingredients and chemistry only dimly understood by the majority of the general populace. In contrast, the mask operates as a complex and evocative visual signifier. Covering much of the face, it also erases individuality and insists on visible collective responsibility. Vaccination also, when administered across a general population, asserts a shared biological experience in a way that some may find unsettling, even to the point of eliciting what Priscilla Wald describes as an “ontological tremor” (2008, 53). As she notes early in her work on outbreak narratives, “[T]he biological aspect of community articulated in the idea of herd immunity makes any catastrophic illness a communally transformative event at the deeply conceptual (and psychological) level as well as, more explicitly, in social terms” (53). The mask and the vaccine both confront the individual with the reality that our immune systems are interlinked, that we are, as Eula Biss reminds us, “each other’s environment. Immunity is a shared space…” (2015, 163). However, despite immunologists’ call for a more “ecological”^5^ understanding, our sense of the immune system remains deeply tied up in notions of selfhood and individualism that threaten to undermine the aims and objectives of public health unless an alternative immunopolitics grounded in notions of altruism and sacrifice may be fostered in their place.^6^

In many cases, conversations on social media centering on pandemics, vaccines, and personal rights feed off popular culture and are driven by celebrities and so-called influencers who wield a degree of persuasive appeal not typically available to public health professionals. Grounded in affect, these arguments play on the emotions of parents and caregivers while fostering mistrust in the vaccine industry as just one more arm of ultra-capitalist Big Pharma. As such, an understanding of how cultural products influence public health literacy is crucial, as Evie Kendal has argued in a cogent article examining a series of recent viral outbreak films through the psychological lens of Protection Motivation Theory (PMT), attempts to account for how individuals assess and deal with risk in the context of health crises. More needs to be said on whether popular culture erects barriers to effective preparation for vaccination or helps to instill confidence in the general populace. The obvious answer is that it does both; as has been noted elsewhere, “popular culture and biomedicine are constantly informing one another,” playing a central role in the “distribution of knowledge” (Görgen, Nunez and Fangerau 2019, 2). For many of us not trained in medicine, our understanding of the healthcare system in all its complexities is mainly derived from visual media, such as television or film and perhaps increasingly, from documentaries on popular networks like Netflix, or conversations occurring across social media with varying levels of accuracy and evidence-based reasoning.

So in this audio-visual/social media cultural spirit, a question that has been asked before must be asked again: to what degree are pro-vaccination cultural campaigns successful in fortifying public health efforts against misinformation and ideological hesitancy? Films like Steven Soderbergh’s *Contagion* (2011) reinforce vaccine optimism when they show images of people eagerly taking their place in physically distanced lines in order to receive their vaccine against a virus that has tragically cut through their lives throughout the film. Yet the fictional at times paints a more idealistic image than the real, and *Contagion* is certainly invested in a narrative of medical triumphalism,^7^ the depiction of science prevailing over a virus in a manner that particularly showcases American ingenuity. The film effectively occludes the other side of the picture, namely, the people who will not be waiting anxiously for their birth date to be called in the lottery and those who won’t keep their appointment or even force their way to the front of a line. And in the current situation, one should note that those who are hesitant may in fact be more likely to accept a vaccine that is not so heavily politicized, as it has been in the United States.^8^ This article acknowledges the heightened complexity of vaccine hesitancy in relation to SARS-Cov-2. We should be wary of assuming that the usual reasons informing vaccine hesitancy are at work in the current situation. On the contrary, a novel virus brings novel hesitancy that is expanded beyond what we typically encounter, extending to all demographics, age groups, generations, and genders, with polls in early December indicating that women particularly are less likely to accept a vaccine.^9^ Despite being a film heavily invested in the gender politics of infection (as I will discuss later in this paper), vaccine uptake in *Contagion* appears to be widely embraced by men and women unlike and does not face the level of skepticism (despite being produced relatively quickly) that SARS-CoV-2 vaccines are attracting.

Indeed, contemporary outbreak narratives like Soderbergh’s *Contagion* condition us to see the vaccine as the end point of the arc of contagion, marking the eventual containment of an elusive virus and allowing for the resumption of life not so fundamentally different from pre-pandemic routines. The rhetoric around vaccine development as a miraculous response to a virus that resists containment certainly perpetuates these assumptions and may also unhelpfully interfere with other public health directives like social distancing and mask wearing. The assumption these visual narratives reinforce is that a pandemic will awaken each of us to the urgency of vaccination and persuade us to put aside our reservations and anxieties because our fear of the virus and the threat it poses to our way of life exceeds our concerns over new and unfamiliar vaccines. These stories underscore the idea that compliance is the inevitable outcome of quarantine. However, polling and conversations already happening on social media suggest a different reality.^10^ It is entirely possible that *“*Operation Warp Speed” may actually impede acceptance of a vaccine perceived as rushed through its trials for the sake of political expediency.

Paula Treichler’s foundational work, *How to Have Theory in an Epidemic,* attunes us to the set of discursive dichotomies that structure our understanding of disease (1999, 35); although she is primarily concerned with reconstructing the AIDS “text,” her critique applies to an emergent virus like SARS-CoV-2 that, in the paucity of medical information, we interpret through those lenses with which we are most familiar: hence, oppositions like “self and not self,” “virus and victim, guest and host,” “contagion and containment,” “expert and patient” and so on (35). The preponderance of outbreak narratives in popular culture render it almost inevitable that we fall into the categories of thought that *Contagion* reproduces in its almost Manichean approach to the ethics of public health response. Hence, the stock figure of the superspreader becomes a Typhoid Mary of sorts, with the standard immune figure playing a hero (as do public health doctors), while the vaccine itself acquires a transcendent status. As Wald points out, there is a formulaic quality to these narratives that render these tropes almost inescapable (2008, 2); at the same time, the conclusion to her book clearly states that the “the conventions that make the story so appealing, which are derived from genres such as myth and horror, also influence the articulation of the global health problem” (266). The outbreak narrative frames our understanding of pandemics and more specifically, of the role of vaccines. As such, attunement to the representation of both vaccines and vaccine compliance as inevitable is important in public health planning.

Discussions of *Contagion* centre on the film’s scientific accuracy, although, as D.A. Kirby notes, “the concept of ‘accuracy’ is not a stable category when applied to movie science” owing in part to science’s “flexibility” (2014, 20). According to Kirby, film makers long made the mistake of viewing science as “a fixed collection of facts,” a “monolithic entity” making “binary choices between the ‘accurate’ and the ‘inaccurate’” (21). Still, much has been made of the extensive consultations with virologists, epidemiologists, and public health officials carried out in the interest of realism. As a film that has come under scrutiny since the onset of the COVID-19 pandemic, the prescience of the screenwriters in predicting how governments might respond to a crisis of this nature has been consistently lauded. However, more attention to the way the film reproduces basic tropes of the outbreak narrative reveals a certain Hollywood narrative typicality and determinism at work in the resolution of the film. Much like a horror movie, the spectre of the virus is contained at the end as the temporarily disrupted borders between nations and bodies are redrawn and reinforced.

At times, Soderbergh’s film opens up a window into the biopolitics of the public health effort to contain the virus, to which Krumwiede’s panning camera seems to be alluding in his final scene as he records the scenes of vaccine distribution overseen by the military. Seen through Krumwiede’s lens, these tracking shots establish the vaccine line up as a militarized space, reminiscent of the “utopia” of the disciplinary society that, according to Michel Foucault’s analysis in *Discipline and Punish *(1975), emerges in times of plague, thereby laying the groundwork for modernity’s development of biopolitics. To a certain extent, the film demonstrates that quarantine allows for the containment of a population that can then be studied, analyzed, and controlled. However, closure in the case of *Contagion* is incompatible with realism and represents a distorting simplification of public attitudes towards vaccination. Far from the docile bodies produced by Foucault’s idea of a disciplinary society, there is considerable evidence that we are far from compliant. Rather than conditioning us to follow unquestioningly the directives of public health authorities, months of lockdown have instead produced a kind of dis-ease with authority, as anti-vaccine protests against social distancing and mask-wearing continue to reverse all efforts to flatten the curve.^11^ As preparations to distribute coronavirus vaccines scale up, public health campaign will have to address this situation with care, making sure to avoid the failures of vaccination campaigns in the past and to meet hesitancy with sound science (where it exists) and compassionate messaging.^12^

Although *Contagion* affords the viewer a fleeting opportunity to see the world through the skeptical point of view of Alan Krumwiede (Jude Law) as he takes on the role of videographer/alter ego for Soderbergh, ultimately the film rejects this perspective by rendering him as a caricature of a self-serving anti-vaxxer profiting from conspiracy theories about Big Pharma to sell his own “natural” alternative of forsynthia. In the end, Krumwiede’s character is an outlier, representing a face of opposition who ends up vilified and exposed towards the film’s end, contained like the virus and restored to the space of abjection. He, like the virus, is the villain^13^ standing in the way of scientific progress, his self-serving contrarianism contrasted with the heroically self-sacrificing scientist, Ally Hextall (played by Jennifer Ehle), who accelerates clinical trials by injecting herself with an experimental vaccine. After this moment, the scene shifts from a laboratory setting to the hospital bedside of her dying father, where, in a tender, humanizing encounter, Hextall deliberately exposes herself to infection in order to put her newly developing antibodies to the test. Like most other Hollywood “disaster” narratives, the film oscillates between poles of good and evil, sin and virtue, as well as self-interest and self-sacrifice. We see this melodrama played out clearly in the sharp contrast between, on the one hand, the Beth Emhoff (Gwyneth Paltrow) superspreader figure, whose marital infidelity somewhat gratuitously but spectacularly facilitates the spread of the virus, and her devoted, inexplicably immune husband (Matt Damon) on the other.^14^

In rather typical fashion, the diseased body of the cheating wife reflects her moral failures in an alignment between the physical and the moral that goes back to the eighteenth century and remains tenaciously at work in our current thinking (Vila 2018). As René Girard averred, every plague story needs a scapegoat, and since the early twentieth century when Typhoid Mary became a household name, women and racialized “others” have filled that role. Evie Kendal observes that in outbreak films, “There is also a tendency to conflate stigmatized behaviours, such as alcoholism, sexual infidelity, sex work and homosexuality with increased disease transmission risk” (2019, 7). Citing *Contagion* specifically, Kendall notes that “it is the secrecy surrounding one infected woman’s extramarital affair that makes tracing the origin of the mutated virus before it spreads impossible” (7). Doubling as both a “fallen woman” and Patient Zero, Beth Emhoff’s character dies shortly after returning from her liaison with her lover (only represented in the film by the voice of the director, Steven Soderbergh, and the character’s corpse), her secrecy belied by the trace of contagion her corpse leaves behind. However, her residual impact dominates the film, which only in the final scene circles back to the original moment of viral transmission before moving further back in time to expose how the deforestation efforts of Emhoff’s multinational corporation created the ideal conditions for the virus to jump from one species to another (in this instance from a bat to a pig to a human). Blame in the final analysis is hence dispersed, as if the virus is a result of human tampering with the earth and, in this case, a product of globalization and capitalists’ wanton destruction of wildlife habitats. In this respect, the film attempts to skirt xenophobic blaming of Asia for triggering pandemics through wet markets.^15^ However, one could also argue that this framing, occurring in the final minute of the film, comes too late and seems almost gratuitous, given the scapegoating of Paltrow’s character through much of the narrative. From her opening cough as she speaks on the phone to her lover, she is marked by conjugal infidelity and sexual transgression, mingling elements of the Oedipal (remembering that the Theban plague was attributed to incest)^16^ with the modern mythical figure of Typhoid Mary.

In contrast to Emhoff’s morally and physically diseased state, her devoted husband is a figure of health, the sole naturally immune character of the narrative. As such he can move through urban spaces largely unchallenged, much like Daniel Defoe's infection-impervious narrator of the *Journal of the Plague Year* (1722). In this early plague narrative, H.F.’s wanderings through the streets of 1665 plague-ravaged London provide a first-hand account of the horrors of the time. An island of immunity, Emhoff's character (played by Matt Damon) is also a witness to the unraveling of the social structure as the crisis escalates, while standing up for those who are victimized (as when he fends off someone trying to steal a woman’s rations). Mitch Emhoff also morphs over the course of the film from a demoralized and disillusioned widower into an exemplar of American individualism and protective fatherhood (on display in particular when he wields a shotgun retrieved from a neighbour's abandoned house to drive off his daughter’s persistent boyfriend). Left to grieve amidst betrayal, his is a complicated mourning—a melancholic, unfinished project that, in conjunction with his natural immunity, deepens his sense of alienation from a society already fractured by the pandemic. His immunity establishes the biological barrier between himself and others that contagion in all other respects works to dismantle. It is a shield that extends to his daughter as well, ultimately becoming integral to the survival narrative of the only two prominent (sympathetic) characters at the centre of the film that do not belong to the world of medicine.^17^

Despite the fierce competition with popular movies like *Harry Potter*, *The Twilight Saga: Breaking Dawn: Part 1*, and the re-release of *The Lion King*, *Contagion* became a domestic and international Box Office hit when it premiered in 2011, grossing $8 million on its first day^18^ and winning the weekend. A possible reason for its popularity and success was that unlike early plague narratives, which end with a disease mercifully losing potency with the change of the seasons,^19^ *Contagion* concludes with a happy ending brought about by the development of a vaccine, which inevitably yields a more satisfying finale than an attenuated virus running out of potency and hosts over time. As such, the vaccine is the true hero of the contemporary outbreak narrative, and in the case of *Contagion,* it is continually aligned with sacrifice. This sacrificial valency appears in the redemptive decision of Dr. Ellis Cheever (Lawrence Fishburne) to give his privileged dose to the young son of the CDC custodian, who had drawn a lottery date towards the end of the vaccination calendar. Similarly, the spontaneous decision of the WHO epidemiologist (played by Marion Cotillard) to give up her freedom in exchange for a real vaccine rather than the placebo that had been arranged for her captors, involves a voluntary sacrifice. In the latter case, Dr. Orantes returns to the rural Chinese village in which she had been held captive upon realizing the case of intranasal spray vaccines for which she has been exchanged is a WHO ruse in a grim metaphor for the impersonality of science that the film regularly interrogates. Over the course of her subplot, Orantes metamorphoses from an all-business epidemiologist clinically tracking cases into a (albeit captive) teacher intimately involved with her students *as people*.^20^ In both cases, the vaccine is bound up with moral decisions that humanize both the scientists and those typically marked as “other” in outbreak narratives of the twenty-first century.

The vaccine as such becomes a gift, but one that, linked with redemption, *seemingly* exists outside the circle of debt and repayment theorized by the likes of Marcel Mauss and Jacques Derrida.^21^ However, as both theorists have shown, all gifts make claims on their recipients, while benefiting the giver: in the case of *Contagion,* the gift confers on the benefactor an unquestionable moral status and is presented as one that cannot be declined. At the same time, this gift strengthens the social bond, in that sense performing the function identified by Mauss of promoting cohesion and stability. This gift in the film is one that cuts across class, as in the example of the CDC director and the custodian’s son, and across race, as in the just cited example and in the case of the WHO epidemiologist and the Chinese community to which she returns as a willing hostage. It is a gift of possibility and of the self that overcomes the boundaries between individuals but in a way that replaces the paradigm of infection with one of prevention, if not healing.^22^

In the popular imagination, immunity is intrinsically bound up with the sacrificial. Popular action-adventure games like *The Last of Us* (Sony 2013) and films like *The Girl With All the Gifts* (2016) represent young people whose imperviousness to the outbreak (in the case of each, a fungal pandemic caused by *ophiocordyceps*) renders them exceptional bodies offered up for the salvation of the collective good. The British post-apocalyptic film directed by Colm McCarthy begins with a sympathetic teacher reading aloud the myth of Pandora to a captive class of “second-generation” children who share the appetites of the fully infected, zombie-like beings referred to as “Hungries” but also retain control of their mental faculties (and, as the teacher is anxious to demonstrate, the capacity for imagination and empathy). Although the film’s title ostensibly refers to the protagonist’s immunity as a “gift” to be bestowed on the remaining survivors, Melanie’s refusal of self-sacrifice (signalled by her release of fungal spores into the atmosphere at the film’s end) circles back to the story of Pandora and thereby invests the idea of “gift” with an entirely different significance. The release of the spores signifies the eclipse of humanity by the new hybrid being represented by Melanie, as her teacher begins the film as she began, reading to children, albeit now enclosed within a controlled environment for her own protection.

While both gaming and film fungal outbreak narratives resist the notion that the individual must be sacrificed for the good of the many, the immunity-producing vaccine in films like *Contagion* positions the idea of sacrifice somewhat differently. In repeatedly making of the vaccine a gift, the film underscores collective responsibility while affirming the heroism of individuals, as in the case of Matt Damon’s portrayal of the “Great American Dad.” The moral system of the film foregrounds the crises of conscience experienced by medical practitioners torn between loyalty to family and responsibility to the public. The sacrificial logic of vaccination transcends gender categories, as men and women alike embrace their responsibility to society’s most marginalized in ways that cut across national, ethnic, and class boundaries. However, the women doctors’ sacrifices are by far the most striking with Dr. Ally Hextall’s spontaneous decision to test the vaccine on herself and expose herself to infection credited with saving untold millions of lives. The transgressions of Patient Zero are in this sense redeemed by the selflessness of a woman doctor who, instead of accepting the recognition and accolades that are her due, in her final act deposits the now neutralized virus in a high security bio-containment freezer that also houses the SARS and H1N1 viruses. In this sense, she is the inverse of a Pandora figure, putting the lid back on a fatal danger that Emhoff’s company helped unleash and she herself helped spread.^23^ Suggestively, the image of the sealed lab freezer subsequently gives way in the next scene to that of a neatly wrapped present, Mitch Emhoff’s prom night gift to his daughter, in an evocative match cut that builds a thematic connection between the two scenes and further solidifies the vaccine’s connection with the gift. At this point in the film, the tense pulse of the soundtrack subsides into a reassuring baseline and eventually into the diegetic music of the private prom at Emhoff’s house made possible only by the persistent boyfriend’s proof of vaccination. The completion of the immune husband’s mourning for his Patient Zero wife coincides with the film’s ending, as his daughter’s celebration downstairs marks an eventual return to normalcy, a melodramatic celebration of reunion that holds out promises of healing for the next generation.

In their critique of “contemporary approaches to reducing vaccine hesitancy,” Emily Harrison and Julia Wu write: “For vaccination, or any pre-emptive measure, to take root across a diverse public, we expect that constructs of care and social solidarity must be as strong as the desire to protect and determine our own futures and our faith in the possibility of being saved. We must practice these constructs in word as well as deed. This likely means a re-imagination of cultures of public health, in which the ideal of social solidarity is granted enough power to infuse and shift our guiding ethical constructs” (2020, 325). Films like *Contagion* reinforce ideas of social solidarity and interconnectedness; indeed, the resumption of the hand shake at the end of the film is a deeply symbolic gesture that models the elimination of danger, the restoration of normalcy, and openness to the other, all made possible by vaccination. Whereas Soderbergh’s lens renders physical contact and fomite-retaining surfaces horror inducing and abject,^24^ the ending works hard to neutralize those fears by rendering touch and social practices like handshakes (relatively) innocuous once more. In shaking hands with the custodian father of the child to whom he has just given his vaccine, the CDC director, Dr. Cheever, traces the practice to the culture of the “Wild West,” but historians’ claim that it evolved as a democratic gesture as an alternative to the scraping deference of a bow or curtsy better captures the leveling effect of the moment in asserting shared humanity (Zuckerman 2000). In this context, Dr. Cheever’s sacrifice of his own vaccine, much like the handshake, allows for the bridging of class (and racial) divides in a moment typical of the visual sensibility of the film that almost seems to capture in freeze frame exchanges like this between characters.^25^

In foregrounding the sacrificial, the film rejects the culture of individualism that drives anti-vaccination movements and models an ethos of care in its place. In this regard, the film seeds ideas in the public imagination that may in fact be conducive to public health. However, this ethos is itself highly contingent on a successful vaccination campaign, which, given our current climate of vaccine hesitancy, one should certainly not take for granted. In the end, the contemporary outbreak narrative conditions us to see the vaccine as the inevitable resolution of a pandemic. The reality of a sizeable segment of the population opting out of vaccination and thus thwarting public health efforts is not considered in the interest of closure. But what if there is no closure? What if, rather than a single outlier neutralized by his duplicity, hesitancy remains pervasive? What if vaccine optimism is itself an illusion, fed by cultural products like *Contagion,* that prevent us from preparing adequately for a very different climate once a vaccine becomes available? What if the twelve million followers of Alan Krumwiede, whose continued loyalty is ominously referenced in relation to his ability to raise bail and wander at large again as a vocal critic of the biopolitical operation of the vaccine campaign, were in fact to act upon his suggestion that the vaccine is unsafe? COVID-19 invites us to imagine a sequel to *Contagion* in which a thwarted vaccine campaign has allowed the virus to become endemic, flaring up at intervals in pockets of the global population. This would perhaps resonate more closely with reality and possibly serve as a warning to the vaccine hesitant although communications experts disagree on whether optimistic or negative messaging is most effective in promoting public health objectives. However, in this instance, a story of virus containment affords reassurance at the price of the real. This is not to say that a film has any responsibility to do anything less or more than this, but from a public health perspective, it may dilute some of the urgency of a pandemic by pushing a narrative of vaccine determinism.

In reference to the “outbreak narrative's tendency toward epidemiological containment of and solution to newly emerging diseases,” Bill Albertini (2008) notes that certain “narrative energies” in these works actively resist closure, perhaps taking their cue from the ongoing AIDS epidemic, which he characterizes as the “central uncontained outbreak in the cultural imaginary” (“Containment” np). In these works, closure and containment exist simultaneously, with narrative openness serving as a reminder of bodily fragility and vulnerability. Although *Contagion* works hard to reinforce a sense of containment, by concluding with what should sequentially be the beginning of the narrative with the destruction of the infected bat’s habitat, it echoes scientists’ warnings that future pandemics are inevitable if the land-clearing practices represented by the multinational conglomerate of Alderson continue at their current pace. As such, the sense of containment the film offers is applicable only to the fictional virus,^26^ MEV-1, and is temporary and contingent. That said, the narrative of containment by vaccine is absolute, and this in itself is cause for considerable concern.

Ultimately the film’s didactic message coalesces around an ethic of sacrifice that sidesteps the politics at work in the American scene in particular, eliding the debate over agency and individual autonomy becoming more prominent as the COVID-19 pandemic continues. The ethic of sacrifice papers over some of the more problematic gender representations; in celebrating the voluntary self-sacrifice of scientists whose actions allow for the purging of contagion, the film invites us to forget that a character has been sacrificed at the outset of the narrative, set up as a straw (wo)man upon whom blame can comfortably rest as contagion spreads across the globe and other characters—many of them women—step up with strong displays of selflessness. It is striking that a film so steeped in moral messaging would, in fact, evade the ethical questions surrounding vaccination, but this could very well have everything to do with the fact that mainstream society might see this conclusion as a given (hence Heidi Larson’s observation that no real outreach or attempt to see the other side of the debate is ever attempted).^27^ For despite its nuances and moral complexity, *Contagion* is a film that relies on vaccination as a plot device—a modern-day *deus ex machina* made possible by science—rather than as an extension of its sustained reflection on the vulnerabilities that connect us all.

In these unusual times, the humanities as a discipline invites us to consider the people affected by the pandemic rather than simply the disease and as such has a significant role to play in conversations around vaccine hesitancy in the coming months or years as public health organizations address the formidable challenges to fostering confidence in a COVID-19 vaccine. Will SARS-CoV-2 be as amenable to control as the fictional virus consigned by Ally Hextall to the laboratory freezer at *Contagion’*s end? Will the general population across the globe accept vaccination should one emerge? Time will tell, as we dwell in the realm of the speculative at a moment when a new and rapidly mutating virus puts medical science and collective responsibility to the test. As COVID-19 compels us to examine anew fictional products of popular culture that shape our understanding of public health, one important fact remains certain: we must be wary of how our assumptions about the arc of this and future pandemics are informed by the typical determinism of the virus thrillers we consume.

## Endnotes

^1^ As do scientists involved in the Vaccine Confidence Project. See Philip Ball’s May 13 2020 piece, “The Anti-Vaccine Movement Could Undermine Efforts to End Coronavirus Pandemic, Researchers Warn.”

^2^ For background on the twentieth-century debates, see James Colgrove’s work on the politics of vaccination (*States of Immunity: The Politics of Vaccination in Twentieth-Century* America) and Elena Conis’s *Vaccine Nation: America’s Changing Relationship With Immunization.*

^3^ See Sarah Heath’s article in *Patient Engagement.*

^4^ At the time of writing this article, Pfizer and Moderna had both announced that their vaccines are 90% and 95% effective, respectively.

^5^ See Alfred Tauber in particular for a corrective to the “defensive” model of immunity. In addition, the anthropologist, Emily Martin, has been addressing individualistic and gendered constructions of the immune system since the 1990s from an anthropological perspective.

^6^ Indeed, as Mark Davis and Davina Lohm have observed in in recent work on the 2009 H1N1 pandemic, “contemporary immunopolitics are individualized and have therefore abandoned the collective mobilization of public life in relation to pandemic threat. Politically and biologically, immunity is foremost a method of signifying how self is distinguished from other in the process of detecting and destroying pathogens” (2020, 115).

^7^ As Priscilla Wald writes, outbreak narratives are typically stories of “tragedy and triumph, horror and salvation” (2008. 56).

^8^ Having dismissed the appeals of scientists and public health officials for the last year, the former Trump administration’s embracing of science for political expediency is understandably a cause for alarm, putting those who generally favour vaccination in the awkward and unfamiliar position of now questioning its science. Some anti-vaxxers in the United States also find themselves called to vaccinate by someone they look to as a leader, in a disorienting disruption of the ideological positions that typically align with the divergent and irreconcilable world views of the right and the left.

^9^ A National Geographic and Morning Consult poll released on December 7 filled many with consternation when it revealed that fully one quarter of respondents identifying as women indicated that they were unlikely to take a coronavirus vaccine when one came available. This news shook the optimism of those triumphantly celebrating the Pfizer and Moderna vaccines as unprecedented breakthroughs in the world of vaccinology.

^10^ Polls that Professor Joshua Greenberg and his research team at Carleton University in Canada conducted in the spring of 2020 suggested that the curve of vaccine hesitancy had not in fact been flattened and that the endeavour to foster vaccine confidence in the general public will pose more of a challenge than popular visual narratives like the one cited above might suggest. Discussed in this university news item: https://newsroom.carleton.ca/2020/new-carleton-research-finds-covid-19-not-flattening-vaccine-hesitancy/.

^11^ Although polls released in December of 2020 shortly before the Pfizer vaccine received FDA approval suggest that confidence in the science of the mRNA vaccine has increased in recent months, they nonetheless indicate that considerable skepticism remains, with one in four of the women polled purportedly identifying themselves as “unlikely” they would accept a vaccine. Indeed, as mentioned above, the particular conditions under which SARS-Cov-2 vaccines are produced invite us to reconsider what it means to be hesitant.

^12^ Madison Powers and Ruth Faden have addressed these failures at length in *Social Justice: The Moral Foundations of Public Health* (2006), which establishes the promotion of well-being (and remediation of existing inequalities) as central to a social justice-oriented program of public health. In a thread on Twitter and articles published in *The Atlantic,* Julia Marcus has called for more empathetic public health outreach and harm reduction infused with empathy. https://www.theatlantic.com/ideas/archive/2020/05/no-one-telling-americans-how-reopen-their-lives/612172/.

^13^ In her account of how viruses are ascribed agency and malevolent intent in outbreak narratives from the 1990s onward, Priscilla Wald writes, “Nothing better illustrates the reluctance to accept Nature’s indifference toward human beings and the turn from the ecological analysis in accounts of emerging infections of all varieties than the seemingly irresistible tendency to animate a microbial foe…Scientists emphasize the microbes’ lack of conscious agency. But the animation of the microbe invariably surfaces during the course of these accounts” (2008, 42).

^14^ Interestingly, Mitch Emhoff as a lay person is the foil for Krumwiede; he is a science accepting, pro-vax everyman able to move freely in public without the make-shift hazmat suit of the latter.

^15^ For more on xenophobia and virus narratives, see Bill Albertini’s “Geographies of Contagion.” Heather Schell also notes that outbreak narratives of the 1990s typically take Africa as the point of origin for pandemics, whereas Asia has increasingly become the focus since SARS, while Priscilla Wald’s *Contagious* addresses Sinophobia in her introductory chapter to *Contagious.*

^16^ Wald characterizes Oedipus as the original “superspreader” in her discussion of transgression and divine displeasure in early plague narratives (2008, 15–17).

^17^ The CDC custodian and his son are two other characters unaffiliated with public health but are comparatively peripheral to the narrative, while Alan Krumwiede is clearly cast as the villain of the film.

^18 ^See Pamela McClintock’s “Box Office Report: Steven Soderbergh’s ‘Contagion’ Winning Weekend Race” (September 10, 2011). *The Hollywood Reporter*. Retrieved June 28, 2012.

^19^ Examples include Daniel Defoe’s *Journal of the Plague Year,* Albert Camus’s *La Peste,* and Charles Brockden Brown’s *Arthur Mervyn.*

^20^ Another example of medical altruism is demonstrated by the contact tracer, Erin Mears, who, having contracted MEV-1, finds herself in the very emergency treatment centre she had helped establish and gives her coat to a bedmate as a dying act of charity.

^21^ See Marcel Mauss, *The Gift* and Derrida, *Given Time. I. Counterfeit Money*.

^22^ One might also look to the 2007 film adaptation of *I am Legend* for a similar resolution in the Christ-like gift of his blood that Robert Neville proffers to the survivors of the zombie/vampire plague as a kind of vaccine (that the film gets the science of vaccination dramatically wrong is another matter).

^23^ As LA Alfonso argues, “the shot of freezers containing ‘secure’ samples of deadly viruses is followed by an image of a silver bow on a gift-wrapped present. The shape and silver of the bow suggest intentionally but subliminally an opening of a Pandora’s Box in a future scenario.”

https://vaxxers.net/2020/12/05/contagion-reinfection-in-the-year-2020/.

^24^ I am indebted to an anonymous reviewer for this observation.

^25^ I have discussed the racial implications of this exchange in a previous article on vaccine rationing.

^26^ A virus that is apparently based on SARS.

^27^ See opening comment of this article for context.
